# Integrating Tobacco Cessation Quitlines Into Health Care: Massachusetts, 2002–2011

**DOI:** 10.5888/pcd9.110343

**Published:** 2012-07-26

**Authors:** Donna D. Warner, Thomas G. Land, Anne Brown Rodgers, Lois Keithly

**Affiliations:** Author Affiliations: Donna D. Warner, Multi-State Collaborative for Health Systems Change to Reduce Tobacco Use, Rhinebeck, New York; Thomas G. Land, Lois Keithly, Department of Public Health, Boston, Massachusetts.

## Abstract

QuitWorks is a Massachusetts referral program that links health care organizations, providers, and patients to the state’s tobacco cessation quitline and provides feedback reporting. Designed collaboratively with all major Massachusetts health plans, QuitWorks was launched in April 2002. In 2010, approximately 340 institutions and practices used QuitWorks. Between April 2002 and March 2011, approximately 3,000 unique providers referred patients and 32,967 tobacco users received referrals.

An analysis of QuitWorks data showed 3 phases in referrals between April 2002 and March 2011: referrals increased from April 2002 through November 2005, plateaued during December 2005 through January 2009, then substantially increased during February 2009 through March 2011. Factors responsible include partnerships with stakeholders, periodic program promotions, hospital activities in response to Joint Commission tobacco use measures, service evolutions, provision of nicotine replacement therapy for referred patients, and electronic referral options. QuitWorks’ history demonstrates that tobacco cessation referral programs can be successfully sustained over time; reach substantial numbers of tobacco users, benefit providers and health care organizations; and contribute to sustainable systems-level changes in health care.

## Introduction

In the efforts to curtail tobacco use in the United States, a clinical practice guidelines goal is that every tobacco user should receive an intervention and be offered treatment at every health care visit (1). Evidence-based tobacco cessation services by telephone, called quitlines, are available throughout North America and are a component of efforts to achieve this goal (2). In the United States, all quitlines are linked through 1 electronic portal —1-800-QUIT NOW — that automatically connects callers to their state’s quitline.

Quitline services are generally funded by state-level tobacco control programs or a mix of state, federal, and private partners. Typically, a quitline counselor will conduct a screening interview and offer 1 or more counseling sessions before, during, and after a quit attempt. Some quitlines also offer free nicotine patches or other medications, self-help materials, and access to Internet resources. Most quitlines conduct evaluation calls after 6 months to assess clients’ quit status (3).

In April 2002, the Massachusetts Tobacco Control Program (now called the Tobacco Cessation and Prevention Program), an initiative of the Massachusetts Department of Public Health (MDPH), launched QuitWorks, a tobacco cessation program through faxed referrals that links health care organizations, providers, and patients to the state’s quitline. The goal was to fill a gap for providers who lacked a consistent resource to which to refer patients who use tobacco. The MDPH and 8 commercial and Medicaid health plans forged a partnership to make QuitWorks universally available and free to any provider in the state and to all residents, regardless of insurance status.

Following Massachusetts’ lead, other states adopted the fax-referral option. By 2009, all 50 US states, the District of Columbia, Puerto Rico, Guam, and all 10 Canadian provinces reported offering these services, although the range of program practices varied widely across locations (State quitline programs, unpublished survey of state quitline referral practices, 2010).

The heart of the QuitWorks program is a simple Health Insurance Portability and Accountability Act–compliant enrollment form completed by the provider, the patient, or both. The patient signs the form and the provider sends it to the quitline by fax or other electronic means. By signing the form, the patient gives consent for the quitline to call and authorizes the quitline to send reports on the patient’s progress to the provider. All referring providers receive 2 reports on each patient referred — 1 within 5 weeks on quitline attempts to reach the patient and the services accepted, and a second 7 months later on the patient’s quit status.

Throughout its history, QuitWorks has primarily received referrals from 4 classes of facilities — hospitals (56.0%), outpatient clinics (14.0%), community health centers (10.1%), and provider practices (8.7%). The remaining 11.3% consists of providers with no organization or groups that do not easily fall into the categories above, such as nursing homes, public housing programs, visiting nurses, or other human service providers.

QuitWorks has steadily grown during its 10 years of operations. Following its launch, the 8 commercial and Medicaid health plan partners conducted a promotion effort in 2002 and 2003, including delivery of QuitWorks kits door-to-door to 4,800 providers. This effort resulted in a substantial increase in referrals. Referrals increased again in 2005 as a result of hospital activity to meet 2004 Joint Commission tobacco measures (4) and expanded promotions to hospitals and community health centers. By 2006, QuitWorks had developed on-site technical assistance and clinical training services needed to integrate QuitWorks into the clinical workflow, medical record systems, and performance reporting. Beginning in 2009, the availability of free starter kits of nicotine patches for QuitWorks patients statewide contributed substantially to a new spike in referrals, as did the implementation of a fully electronic health records system in Atrius Health, a major QuitWorks user. Periodic free e-mail, newsletter, and fax promotions, conducted each year by MDPH and health plan partners, also increased in 2009 and 2010.

QuitWorks now includes many features not available when it was launched in 2002, among them a web site (www.quitworks.org), customized and flexible manual and electronic referral options, enrollment forms, and reports for participating organizations. The availability in 2011 of Centers for Medicare and Medicaid Services (CMS) incentives for meaningful use of health information technology (5-7) has accelerated the trend among providers to use QuitWorks electronic referral options. Privacy, consent, and security issues for all QuitWorks innovations have been addressed with the assistance of expert consultants and cleared by provider, health plan, public health, and legal departments.

## Analysis of QuitWorks Referral Patterns

QuitWorks has tracked the number of referrals and referring providers since the program’s inception. In addition, all facilities are classified in the QuitWorks database into 6 categories (health care, health plans, human service organizations, worksites/unions, professional or health care associations, and other), which are further classified by health care type (medical, dental, behavioral health, nursing home, wellness programs, and visiting nurse association), facility type (hospitals, community health centers, outpatient clinics or systems, and provider practices), and specialty (eg, obstetrics-gynecology, pediatrics). This allows the QuitWorks team to plan operations, establish goals, and analyze performance across similar types of institutions.

To better understand trends in referral rates to QuitWorks and influencing factors, we conducted a joinpoint analysis of the referral dates. Published by the National Cancer Institute, joinpoint software (National Cancer Institute, Bethesda, Maryland) is used to detect underlying trends in time-based data (8). The “joinpoints” are those points in time where a previous trend ends and a new one begins. In this analysis, we used the number of monthly referrals as the basic input data to the joinpoint software, while the basic output was the annual percentage rate change in referrals during each segment or time phase. The analysis used a log linear model to predict monthly observations. The minimum number of joinpoints was set to 0 and the maximum was set to 5. The minimum number of observations on either side of a joinpoint was set to 6. Models were selected by repetitive permutation tests, and significance levels were adjusted with a Bonferroni correction. Significance tests are based on a Monte Carlo simulation to determine the frequency with which events occur.

## Insights into QuitWorks Referrals and Their Impact

**Figure 1:**
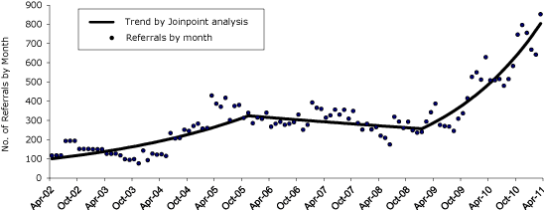
Number of QuitWorks referrals, by month and trend by joinpoint analysis (8), April 2002 through March 2011.

Between April 1, 2002, and March 31, 2011, 32,967 tobacco users received referrals from thousands of providers statewide. Our analysis suggests that QuitWorks has had 3 phases of referrals (Figure 1).

During the first phase, from April 2002 through November 2005, monthly referrals increased from approximately 100 to approximately 325, an annual percentage change (APC) of 38.3% (*P* < .001). During the second phase, from December 2005 through January 2009, the number of referrals to QuitWorks did not change significantly (APC = −7.6%, *P* = .30). However, in February 2009, referrals to QuitWorks began to substantially increase (APC = 69.2%, *P* < .001) during the next 2.25 years. QuitWorks received 3,909 referrals in 2009, 7,067 referrals in 2010, and approximately 9,000 in 2011. The program is on pace to receive approximately 10,000 referrals in 2012. With limited funding to promote the quitline directly to consumers through a media campaign, QuitWorks provider referrals have become the primary source of quitline clients. Since November 2009, provider referrals have accounted for approximately 80% of total client volume of the Massachusetts quitline.

Our examination of referral patterns also suggests that QuitWorks has become an integral component of health care delivery over time, in part because major users have begun to incorporate referrals into their electronic health records. In addition, reflecting the evolution of the program, the sources of referrals have varied over time.

### Integrating QuitWorks into health care delivery

An increase in referrals built on large numbers of providers using QuitWorks just once or twice cannot be taken as evidence of integration into the health care delivery system. However, an increase in the average number of referrals per institution does document a change in practice patterns. This is the pattern seen for the top 10 referring providers and institutions during the third referral phase (February 2009 through March 2011). In this phase, the average number of monthly referrals for these institutions increased by 117%, from 14.4 to 31.2. At the same time, the proportion of total referrals accounted for by the top 10 referring institutions decreased from 54% to 37%, indicating that many new providers were trying QuitWorks even as the top referrers were increasing their average number of monthly referrals. In 2008, 704 individual providers referred patients to QuitWorks. By 2010, 1,919 individual providers referred patients to QuitWorks.

### Integrating QuitWorks into electronic health records

In 2008, QuitWorks was among the last paper-based systems still working with the Harvard Vanguard Medical Associates (HVMA) network. HVMA is a 17-site clinic system serving 325,000 adult patients under the large Atrius Health system umbrella. The QuitWorks paper-based system no longer fit within the streamlined HVMA workflow and required substantial HVMA staff time to manually assign patient identification information to QuitWorks referral forms and reports.

**Figure 2:**
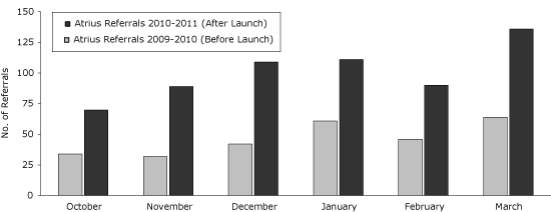
QuitWorks referrals 6 months before launch of electronic referrals (October 2009 through March 2010) and the same period 1 year later, 21 Sites, Atrius Health.

In 2010, Massachusetts launched a fully electronic version of QuitWorks in partnership with the entire Atrius Health/HVMA 21-clinic system, using an interface program that accepts referrals from any electronic health record with patient medical record identification. The interface program also has the capability to transmit feedback reports electronically to the referring provider organization. Once the fully electronic referral system was available, total referrals from Atrius Health to QuitWorks increased markedly. Before the electronic referral launch, Atrius made 279 referrals from October 2009 through March 2010. After the launch, Atrius made 605 referrals during the same 6-month October through March period (Figure 2). October 2009 through March 2011 was a period of increasing referrals to QuitWorks overall. However, the rate of increase of referrals from Atrius sites exceeded the rate of increase from all non-Atrius sites (117% vs 43%). Increases in referrals following implementation of electronic systems may vary because of many factors, including seasonality in quit attempts, a decline in the number of tobacco users over time, or other reasons.

**Table:** 

Month	Atrius Referrals	

	2009-2010 (Before Launch)	2010-2011 (After Launch)
**Oct**	34	70
**Nov**	32	89
**Dec**	42	109
**Jan**	61	111
**Feb**	46	90
**Mar**	64	136
**Total**	279	605

### Assessing changes in referral sources

Our analysis of referrals trends shows that different types of institutions drove the growth of QuitWorks in each of the 3 periods. Hospitals always have been an important source of referrals, but the proportion of referrals coming from hospitals peaked during the second period, and use by practices expanded in the third period.

The third referral period was marked by an expansion of use to 477 practices, hospitals, clinics, community health and social service providers, and approximately 3,000 unique providers. The proportion of referrals from practices increased in this period from 2% to 13% in response to repetitive low-cost promotions announcing the availability of free nicotine patches, while the proportion of referrals from hospitals declined from 63% to 52%.

## Lessons Learned and Implications for the Continued Expansion of Quitlines

Quitlines have been widely adopted in North America and worldwide, and an indicator of their success is the 269% rise in the median number of referrals, including fax referrals, in the United States and Canada, from health care providers from fiscal year (FY) 2005 through FY 2009 (3). However, the overall reach of quitlines remains low, at approximately 1%, even though some states with media promotion or nicotine patch giveaways have boosted reach to nearly 10% (9). Awareness of quitlines and their use by physicians, compared with potential use, also remain low (10). The dilemma facing many quitlines and their state funders is how to increase reach to justify funding levels and, conversely, how to obtain sufficient levels of both public and private funding to both create and meet demand. The integration of quitlines into the health care system may provide a partial solution to this dilemma especially if referral programs evolve, as they have in Massachusetts, into fully electronic referrals that conform to health information technology referral protocols.

The availability of fax and electronic referral quitline programs can effectively advance systematic and sustainable tobacco use interventions in health care. Quitlines are a strategy poised to reach many tobacco users, including subpopulations in which disparities exist in tobacco use or tobacco cessation. Emerging (11-13) and anecdotal evidence (unpublished survey of Massachusetts health care providers, May 2011) suggests that the availability of referral programs can aid identification of tobacco users and brief interventions that precede referral. During the next several years, as health care reform takes effect and more people become eligible for services, the value of incorporating low-cost quitlines as an adjunct to provider counseling and intervention will likely increase.

Best practice examples in the field must continue to be identified, documented, and disseminated through the Centers for Disease Control and Prevention (CDC). The timing is right to make progress now. CMS meaningful use core measures (14,15) and new Joint Commission tobacco use measure sets (16) are likely to influence health care providers to seek linkages to community resources to address tobacco use effectively.

The Massachusetts experience suggests a new strategic direction for state tobacco control programs and their quitlines. As a result of QuitWorks, tens of thousands of tobacco users have received interventions, both in health care settings and through the quitline. By integrating electronic referral to quitlines into routine care, tobacco control programs can extend their impact to reduce tobacco use, reduce risks for chronic disease, improve health, and potentially reduce health care costs. Lessons learned from the QuitWorks experience, as described in the themes discussed below and listed in the Box, may help other state-based quitlines meet the goal that every tobacco user should receive an intervention and be offered treatment at every health care visit.

### Build strong partnerships with quitline users

The sustained engagement of all major commercial and Medicaid health plans with QuitWorks and their endorsement and promotion of the program have been invaluable in reaching thousands of providers each year. In addition, QuitWorks promotions (chiefly inexpensive messaging delivered through partners) have contributed both to an expanding referring provider base and increasing integration into health care delivery.

### Focus on systems change

QuitWorks goals have increasingly focused on sustainable health care delivery systems to support provider behavior change. This focus, exemplified by embedding referrals in electronic health records, may resolve frequently acknowledged barriers to effective tobacco use interventions, including high turnover rate among staff and the loss of champion(s) in the facility or practice, and increase the delivery of tobacco dependence treatment interventions (17,18). The federal Health Information Technology (HITECH) program has accelerated the adoption of electronic health records (19). Given the results from Atrius Health, a growing demand for electronic referral options appears likely.

### Present QuitWorks in the context of quality improvement goals

Another QuitWorks feature is flexible reporting capability. Future QuitWorks’ health plan partners and facilities will probably need reports tailored to their performance reporting requirements. QuitWorks routinely provides reports on patient engagement, services provided, and outcomes, and offers multiple electronic options. This level of responsiveness and customization has strengthened the bond between QuitWorks and its partner agencies. For example, QuitWorks reviews data and report formats periodically with health plans and providers to identify new needs or improvements.

The history of QuitWorks suggests that, when designed properly and integrated into a system of patient care, similar programs might reach significant numbers of tobacco users, while also benefitting providers and health care organizations. Our analysis of trends in referral sources shows that QuitWorks is being integrated into health care delivery, and our findings suggest that QuitWorks has implications for quitlines nationwide.

**Box. QuitWorks: Lessons Learned**

Create a strong partnership with all leading health plans in the area.

Have partners endorse a universal program that applies to any provider and any patient, regardless of insurance status.

Offer free nicotine replacement therapy to clients referred to the quitline by health care providers, especially if the therapy is not otherwise available.

Work with health care providers to promote the program through word of mouth. Encourage participating health care providers to showcase the program at conferences, at professional association meetings, and in other venues.

Identify best opportunities to initiate the QuitWorks program among various types of providers — practices, hospitals, community health centers, other clinics — on the basis of their readiness and capacity to adopt and sustain a quitline referral program.

Build capacity to provide technical assistance to large health care systems to help them integrate referrals to the quitline into their workflow and electronic health records.

Build capacity to share data and reports with health care providers, their institutions, and health plans.

Focus on building capacity for electronic referrals and data exchange between providers and the quitline.

Track and capitalize on health care policy changes or payment reforms that require or create incentives for providers to perform tobacco use interventions.
